# Supplementary material for: Allostery Beyond Amplification: Temporal Regulation of Signaling Information
Source: ArXiv. 2026 Jan 7:arXiv:2601.01850v2. Preprint. [Version 2] (PMC12803326)
Supplement: Supplement 1 [file NIHPP2601.01850v2-supplement-1.pdf]

SUPPLEMENTAL INFORMATION TO "ALLOSTERY BEYOND AMPLIFICATION: TEMPORAL REGULATION OF SIGNALING INFORMATION"

In this Supplemental Information (SI) we explain the chemical master equation (CME) for the allosteric model used in the present study. From the set of chemical reactions in Fig. 1 in the main text, we construct (in SI Sec. A) the chemical master equation (CME) that explains how the counts of each chemical species in the allosteric model change in time. Later (SI Sec. B) we show how we solved the CME in order to produce the graphs in Figs. 3 and 4 and give more details of how the metrics presented there were calculated.

Appendix A: The chemical master equation for allostery model

Chemical kinetics models are often described using average quantities derived from mass-action laws or through a purely thermodynamic framework. However, accurately capturing information transfer via allostery *requires* accounting for the stochastic time evolution of the system. To achieve this, we implement a CME for the reaction network illustrated in Fig. 1. The modeled reactions, along with their stoichiometry, parameters, and kinetic rates are fully detailed in Table A1.

First, we enumerate all possible states of the model by assigning a unique index i to each combination of the model variables $(\sigma_A, \sigma_B, P, S)$. To accomplish this, we need to set maximum counts for S and P, which we denote by I_S and I_P, respectively. The index i for a given combination of $(\sigma_A, \sigma_B, P, S)$ is then defined as:

$$i(\sigma_A, \sigma_B, P, S) = \sigma_A(2I_P I_S) + \sigma_B(I_P I_S) + P I_S + S. \tag{A1}$$

To recover the values of σ_A, σ_B, P, and S from a given index i, we define the following inverse mapping functions:

$$s_A(i) = \left\lfloor \frac{i}{2I_P I_S} \right\rfloor, \tag{A2a}$$

$$s_B(i) = \left\lfloor \frac{i}{I_P I_S} \right\rfloor \mod 2, \tag{A2b}$$

$$s_P(i) = \left\lfloor \frac{i}{I_S} \right\rfloor \mod I_P, \tag{A2c}$$

$$s_S(i) = i \mod I_S. \tag{A2d}$$

Here, $x \mod q$ denotes the remainder when x is divided by q, and $\lfloor x \rfloor$ represents the greatest integer less than or equal to x. We use the notation s_A, s_B, s_P, and s_S to indicate that these are functions mapping the index i back to the model variables, distinguishing them from the raw values σ_A, σ_B, P, and S. In particular, when referencing a state by its index i, this indexing scheme ensures that each function $s_A(i)$, $s_B(i)$, $s_P(i)$, and $s_S(i)$ will return the corresponding value of the model variables (σ_A, σ_B, P, and S) for that indexed state. That is, if a state is indexed by i, then $s_A(i) = \sigma_A$, $s_B(i) = \sigma_B$, $s_P(i) = P$, and $s_S(i) = S$.

With this indexing framework in place, we construct the CME with two elements. The first is the probability vector, which we represent as a row vector $\bar{\rho} = (\rho_1, \rho_2, \ldots, \rho_I)$ where ρ_i is the probability of the system being in state i and I is the total number of modeled states, $I = 8I_S I_P$. As we want to observe the system's time evolution we write $\bar{\rho}(t)$.

The second is the propagator matrix, whose construction is as follows. For each state, indexed by i, we identify all potential resultant state indices (j), which are determined by changes in state indicated in the central columns of Table A1. The rate matrix $\mathbf{\Lambda}$, composed of elements λ_{ij}, is then defined such that λ_{ij} represents the transition rate (specified in the rightmost column of Table A1). The complete rate matrix depends on all the kinetic parameters of the model, which we will collectively denote by θ, making each element a function of these parameters: $\lambda_{ij}(\theta)$. To simplify notation, we write $\mathbf{\Lambda}_\theta$ for the matrix. This rate matrix is then used to construct the propagator matrix $\mathbf{G}_\theta$ with elements $g_{ij}(\theta)$ defined as

$$g_{ij}(\theta) \equiv \begin{cases} -\sum_k \lambda_{ik} & \text{if } i = j \\ \lambda_{ij} & \text{otherwise} \end{cases} . \tag{A3}$$

It is important to note that for each line (or state) i, there are only up to 15 possible reactions (corresponding to the reactions listed in Table A1), thus only up to 15 non-zero λ_{ij} out of I entries in that row, making $\mathbf{G}_\theta$ a sparse matrix.

TABLE A1. Table summarizing the chemical reactions within the minimal allosteric model, as illustrated in Fig. 1. Each line represents one of the reactions, its parameters, and the transition rates between the states before and after the reaction, with δ here representing the Kronecker delta. The central columns represent the change in each of the model variables — labeled as σ_A, σ_B, P, and S — and how they are changed when the respective reaction happens. These values are required to write the rate matrix, as described in SI Sec. A.

Reaction	σ_A	σ_B	P	S	Parameters	Rates
$\emptyset \to S$				$+1$	β	β
$S \to \emptyset$				-1	γ_S	$\gamma_S S$
$A + S \to AS$	$0 \to 2$			-1	$k_{A\text{on}}$	$k_{A\text{on}} S\ \delta^0_{\sigma_A}$
$AS \to A + S$	$2 \to 0$			$+1$	$k_{A\text{off}}$	$k_{A\text{off}}\ \delta^2_{\sigma_A}$
$A^* + S \to A^*S$	$1 \to 3$			-1	$k_{A^*\text{on}}$	$k_{A^*\text{on}} S\ \delta^1_{\sigma_A}$
$A^*S \to A^* + S$	$3 \to 1$			$+1$	$k_{A^*\text{off}}$	$k_{A^*\text{off}}\ \delta^3_{\sigma_A}$
$A \to A^*$	$0 \to 1$				α	$\alpha\ \delta^0_{\sigma_A}$
$A^* \to A$	$1 \to 0$				α^*	$\alpha^*\ \delta^1_{\sigma_A}$
$AS \to A^*S$	$2 \to 3$				α_S	$\alpha_S\ \delta^2_{\sigma_A}$
$A^*S \to AS$	$3 \to 2$				α^*_S	$\alpha^*_S\ \delta^3_{\sigma_A}$
$AS \to A$	$2 \to 0$		$+1$		ν	$\nu\ \delta^2_{\sigma_A}$
$A^*S \to A^*$	$3 \to 1$		$+1$		ν^*	$\nu^*\ \delta^3_{\sigma_A}$
$B + P \to BP$		$0 \to 1$	-1		$k_{B\text{on}}$	$k_{B\text{on}} P\ \delta^0_{\sigma_B}$
$BP \to B + P$		$1 \to 0$	$+1$		$k_{B\text{off}}$	$k_{B\text{off}}\ \delta^1_{\sigma_B}$
$P \to \emptyset$			-1		γ_P	$\gamma_P P$

The time evolution of the states' probability is given by the differential equation [12–14]

$$\frac{\mathrm{d}\bar{\rho}}{\mathrm{d}t} = \bar{\rho}\mathbf{G}_\theta(t)\ . \tag{A4}$$

In the following subsection we show how we set up the kinetic parameters θ, the initial condition $\bar{\rho}$, and how to solve (A4) by employing methods that leverage the fact that we are dealing with a sparse $\mathbf{G}_\theta$ [14]. In short, to construct Fig. 3 we evolve (A4) towards its steady-state ($\bar{\rho}\mathbf{G}_\theta = 0$) while to generate Fig. 4 we evolve (A4) with a varying production rate of S, β. The details are outlined in the following section.

Appendix B: Solving the chemical master equation

To clearly explain the methodologies behind the generation of the main text figures, and consequently the conclusions drawn in our study, this section details the process for solving the CME on the minimal allosteric model. In order to do so we need to define the initial condition, $\bar{\rho}$, and the set of parameters, θ, needed to construct the propagator matrix $\mathbf{G}_\theta$ and solve (A4) for that matrix. However, in the main text we had two different goals, and these translate into two different types of information we want to draw from the CME solution.

The first goal is to find the steady state, $\frac{\mathrm{d}\bar{\rho}}{\mathrm{d}t} = 0$, for a constant generation rate of S, denoted as β, and varying values for the allosteric rate $\xi = \frac{\nu^*}{\nu}$. This generates Figs. 2 and 3 in the main text. We explain the initial condition and the process to find the steady state in SI Sec. B 1.

The second goal, which generates Fig. 4 is to solve the CME for a production rate changing in time. We explain the initial condition and how to solve (A4) and, consequently, obtain the time evolution of the metrics in SI Sec. B 2.

In both cases the degradation rate of S is set as $\gamma_S = 1$, or equivalently $1/\gamma_S$ is the unit of time. In both cases we also show the effect of varying the allosteric rate ξ. We also look at how changes in the ξ rate affect our results. We list all the parameters we used in the main text. In both cases we also explore an equivalent non-allosteric system designed to closely replicate the allosteric kinetics. For it we keep the same model but guarantee that the allosteric states (A^* and, consequently, A^*S) are never reached by the system by fixing the transition to those states as zero.Finally, we explain the metrics used to visualize the steady-state solutions of the first goal and the time evolution of the second goal based on the state probabilities obtained from (A4) in SI Sec. B 3.

1. First goal — steady state of constant β

To construct the initial condition we look into the steady-state of S if the allosteric enzyme were not present. If we restrict the reactions in Table A1 to the first two lines we obtain a birth–death process with respective rates β and

γ_S, thus the steady state would be a Poisson distribution for S with rate $\frac{\beta}{\gamma_S}$. In the cases where β is constant (Fig. 3) we use an initial condition of σ_A in the non-allosteric state A ($\sigma_A = 0$), σ_B in the state of unattached B ($\sigma_B = 0$), initially no product in the system ($P = 0$), and a Poisson distribution with rate $\frac{\beta}{\gamma_S}$ for S. Note that this is done for computational convenience and does not change the steady state.

We evolve the CME using the method described as R-MJP in [14], by using the probabilities described in the previous paragraph as the initial condition $\bar{\rho}(0)$ and then calculating the probability vector at different times $\bar{\rho}(t)$ in intervals of 10, $t = \{10, 20, 30, \ldots\}$. We claim that we have found the steady state when we find a time such that the absolute value of the maximum element in the left-hand side of (A4), $\bar{\rho}(t)\mathbf{G}_\theta$, is smaller than 10^{-12}.

2. Second goal — time evolution for variable β

In the main text, when studying a production rate β that varies in time (Figs. 4, we consider a pulsed production in which the substrate production rate periodically switches between zero and a high value.

In this "on–off" scheme, $\beta(t)$ takes the maximal value $\beta_{\max}$ for a finite portion of each period τ, and remains zero for the remainder:

$$\beta(t) = \begin{cases} \beta_{\max} & \text{if } t \mod \tau > t_i, \\ 0 & \text{otherwise,} \end{cases} \tag{B1}$$

where t_i denotes the duration of the "off" interval within each period. Thus, within every cycle of length τ, $\beta(t)$ alternates between 0 and $\beta_{\max}$, generating a square-wave modulation in substrate supply. An example of this pulsed protocol is shown in the bottom panel of Fig. 4, where we use $\beta_{\max} = 40$, $\tau = 20$, and $t_i = 16$.

To implement this time dependence in the CME, we construct a time-dependent generator $\mathbf{G}_\theta(t)$ using the above definition of $\beta(t)$ while keeping all other kinetic parameters fixed. The initial condition is set as described in the previous subsection, with simulations starting at time $t = -10$ to minimize the influence of initialization transients. In Fig. 4 we present results over four full periods, 4τ.

Because $\beta(t)$ is piecewise constant within each on/off segment, we divide the timeline at the discontinuity points and treat each interval independently, allowing us to apply the same stationary-propagator method used for constant β, referred to as R-MJP in [14].

3. Metrics

Representing the solution of the master equation means obtaining the probabilities of every possible state. In Figs. 2–4 we present the solution in terms of three metrics.

The first metric we use is the mutual information between the states of A and B, defined in (B5). To compute this, we need the marginalized joint probability distribution $p(\sigma_A, \sigma_B)$, which represents the probability of A being in state σ_A and B being in state σ_B. Note that, so far, we have discussed how to calculate the probabilities of the overall states, enumerated by the index i, in the previous subsections (B 1 and B 2). For each state indexed by i, we can determine the states of A and B using the functions $s_A(i)$ and $s_B(i)$, as defined in (A2). Thus, we can express the joint probability distribution $p(\sigma_A, \sigma_B)$ as a sum over all states i:

$$p(\sigma_A, \sigma_B) = \sum_i \rho_i \, \delta_{\sigma_A}^{s_A(i)} \delta_{\sigma_B}^{s_B(i)} \, , \tag{B2}$$

since ρ_i is the probability of being in the state indexed by i, and $\delta_{\sigma_A}^{s_A(i)}$ and $\delta_{\sigma_B}^{s_B(i)}$ ensure that only states with $s_A(i) = \sigma_A$ and $s_B(i) = \sigma_B$ contribute to the sum. Once we have the joint distribution $p(\sigma_A, \sigma_B)$, we can calculate the marginal distributions $p(\sigma_A)$ and $p(\sigma_B)$ by summing over the other variable:

$$p(\sigma_A) = \sum_{\sigma_B} p(\sigma_A, \sigma_B) = \sum_i \rho_i \, \delta_{\sigma_A}^{s_A(i)} \, , \tag{B3}$$

$$p(\sigma_B) = \sum_{\sigma_A} p(\sigma_A, \sigma_B) = \sum_i \rho_i \, \delta_{\sigma_B}^{s_B(i)} \, . \tag{B4}$$

allowing us to then calculate MI_{AB} from (B5) as

$$\mathrm{MI}_{AB} = \sum_{\sigma_A}\sum_{\sigma_B}\left(\sum_i \rho_i\, \delta_{\sigma_A}^{s_A(i)}\delta_{\sigma_B}^{s_B(i)}\right)\left(\log\left(\sum_i \rho_i\, \delta_{\sigma_A}^{s_A(i)}\delta_{\sigma_B}^{s_B(i)}\right) - \log\left(\sum_j \rho_j \delta_{\sigma_A}^{s_A(j)}\right) - \log\left(\sum_k \rho_k \delta_{\sigma_B}^{s_B(k)}\right)\right) . \tag{B5}$$

The other two metrics we are interested in are the expected values of S and P, which represent the average values of these variables over all states. Similarly to how we calculated $p(\sigma_A, \sigma_B)$, we can obtain the marginal distributions for the variables S and P as:

$$p(S) = \sum_i \rho_i\, \delta_S^{s_S(i)} \ , \quad \text{and} \quad p(P) = \sum_i \rho_i\, \delta_P^{s_P(i)} \ . \tag{B6}$$

Once we have these marginal distributions $p(S)$ and $p(P)$, we can calculate the expected values $\langle S\rangle$ and $\langle P\rangle$, defined as:

$$\langle S\rangle = \sum_S p(S)S = \sum_i \rho_i s_S(i) \quad \text{and} \quad \langle P\rangle = \sum_P p(P)P = \sum_i \rho_i s_P(i) \ . \tag{B7}$$

In summary, from the probability distribution ρ_i over states indexed by i — obtained from solving the CME — we can derive all three metrics: the mutual information MI_{AB}, and the expected values $\langle S\rangle$ and $\langle P\rangle$.
